# Functional Landscape of African Swine Fever Virus–Host and Virus–Virus Protein Interactions

**DOI:** 10.3390/v15081634

**Published:** 2023-07-27

**Authors:** Katarzyna Magdalena Dolata, Gang Pei, Christopher L. Netherton, Axel Karger

**Affiliations:** 1 Institute of Molecular Virology and Cell Biology, Friedrich-Loeffler-Institut, Federal Research Institute for Animal Health, Südufer 10, 17493 Greifswald-Insel Riems, Germany; 2 Institute of Immunology, Friedrich-Loeffler-Institut, Federal Research Institute for Animal Health, Südufer 10, 17493 Greifswald-Insel Riems, Germany; 3 The Pirbright Institute, Ash Road, Pirbright, Woking GU24 0NF, UK

**Keywords:** African swine fever virus, virus–host interaction, protein–protein interaction

## Abstract

Viral replication fully relies on the host cell machinery, and physical interactions between viral and host proteins mediate key steps of the viral life cycle. Therefore, identifying virus–host protein–protein interactions (PPIs) provides insights into the molecular mechanisms governing virus infection and is crucial for designing novel antiviral strategies. In the case of the African swine fever virus (ASFV), a large DNA virus that causes a deadly panzootic disease in pigs, the limited understanding of host and viral targets hinders the development of effective vaccines and treatments. This review summarizes the current knowledge of virus–host and virus–virus PPIs by collecting and analyzing studies of individual viral proteins. We have compiled a dataset of experimentally determined host and virus protein targets, the molecular mechanisms involved, and the biological functions of the identified virus–host and virus–virus protein interactions during infection. Ultimately, this work provides a comprehensive and systematic overview of ASFV interactome, identifies knowledge gaps, and proposes future research directions.

## 1. Introduction

African swine fever virus (ASFV) is the etiologic agent of African swine fever (ASF), a highly contagious and often deadly disease of domestic pigs and wild boars. The disease was first described in 1921 in Kenya [1] and has remained endemic in Africa. In 2007, the ASF epidemic emerged in Georgia and subsequently propagated throughout Europe, Asia, and parts of the Caribbean. The lack of available treatments or vaccines against ASFV has resulted in substantial economic losses caused by the ongoing pandemic.

ASFV is a large double-stranded DNA virus of the family *Asfarviridae*. Its genome varies in size, ranging from 170 to 193 kbp, depending on the virus strain, and encodes over 150 known open reading frames (ORFs). Recently, 176 potential novel ASFV-encoded ORFs were discovered [2]. About 68 proteins are incorporated into viral particles [3], and over 100 viral proteins are synthesized in infected cells [4,5]. ASFV primarily infects porcine monocytes and macrophages, although other cell types, such as vascular endothelial cells, hepatocytes, or epithelial cells, can also become infected, particularly during the later stages of the disease [6].

ASFV evolved many strategies for cellular entry and trafficking, formation of virus replication complexes, assembly of virions, and counter-defense of host immune responses, which have been extensively reviewed previously [7,8,9,10,11]. Given such a large number of proteins and the complex morphogenesis and replication strategy, a high number of specific virus–host protein–protein interactions (PPIs) can be expected to be implemented during infection to reprogram the host environment for efficient virus replication and immune evasion. The precise mapping of spatiotemporal virus–host interactions is essential for understanding how the virus exploits the host cell and identifying targets for antiviral drug development.

Over the past decade, knowledge of ASFV–host PPIs has expanded rapidly. Until 2013, only 32 interactions were reported in the scientific literature, while another 99 were reported between 2014 and the end of April 2023. Despite the ever-growing number of reports on novel interactions between ASFV and host proteins, these findings have not been summarized, and the roles of PPIs in the ASFV replication cycle are yet to be discussed. In addition, commonly used virus–host protein–protein interaction database resources such as VirHostNet [12], Viruses.STRING [13], IntAct [14], or BioGRID [15] provide either no or very limited information about the ASFV interactome. Consequently, due to the lack of scientific reviews on this topic and poor curation of the virus–host PPI databases, information on ASFV–host interactions remains scattered, unorganized, and difficult to interrogate.

This paper presents a scoping review of the scientific literature investigating ASFV–ASFV and ASFV–host protein interactions. The focus is on the underlying molecular mechanisms of these interactions and their potential implications in cellular processes and pathways.

## 2. Overview of ASFV–Host Protein–Protein Interactions

The data analyzed in this study were obtained from research articles and include experimentally determined ASFV–host and ASFV–ASFV PPIs, mainly identified through co-immunoprecipitation or yeast two-hybrid methods and, in some cases, mass spectrometry, and validated by co-localization analysis with fluorescence microscopy. To unify the ASFV protein nomenclature employed across multiple studies, we present the protein names as the corresponding genes that encode them.

**Figure 1:**
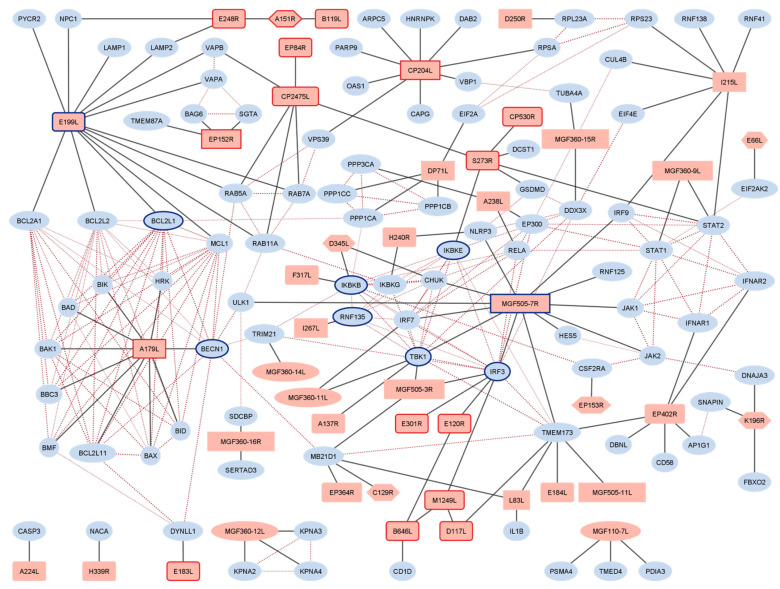
Network representation of the ASFV interactome. Viral proteins are in red, and host proteins are in blue. Essential ASFV genes are highlighted by a red border. Expression kinetics are represented by the label shape; rectangles and round rectangles stand for early and late genes, respectively, hexagons for ambivalent expression, and ellipses for unassigned kinetics. Blue borders indicate the top 5 proteins with potential importance for the network identified with the Bottleneck algorithm (MGF505-7R, BECN1, BCL2L1, IRF3, E199L) or by the Maximal Clique Centrality (MCC) [21] algorithm (TBK1, IKBKB, IKBKE, RNF135, BCL2L1). Red dashed edges indicate interactions among the constituents of the ASFV interactome that were added from the STRING database.

A total of 131 experimentally determined interactions were collected from the literature and are listed in Table 1. Detailed information on identified PPIs and data analysis can be found in Supplementary Table S1 and Supplementary Methods File S1, respectively. The table of interactions was processed with R [16], and a network was constructed with Cytoscape [17] using the RCy3 package [18] as an interface. The network of published ASFV protein interactions alone showed only limited interconnection between the interactors of the different baits (Supplementary Figure S1). After adding the known physical PPIs of host proteins interacting with ASFV proteins from the STRING database [19], the coherent and more complex network shown in Figure 1 was obtained. The number of additional interactions between host proteins by far exceeded the expected number for a random set of genes of the same size, which was indicated by the very low PPI-enrichment *p*-value of 1 × 10^−16^. Network analysis with the Cytoscape StringApp [20] and cytoHubba [21] plugins identified a number of host proteins with potential importance within the network. The detailed data are provided in Supplementary Table S2. The highest Bottleneck score [22] was obtained for the MGF505-7R protein. The Bottleneck algorithm identifies proteins that are important for the topology of the entire network by calculating for each node N_i_ the number of shortest path connections between all possible node pairs that N_i_ is part of. Other proteins with potential significance for the network include IKBKB (interacting with F317L), E199L and its interactor BCL2L1, BECN1 (interacting with A179L), and the MGF505-7R interactor IRF3, to mention the top-scoring interactions.

A Gene Ontology [23,24] term enrichment analysis performed with gProfiler [25,26] showed that the ASFV interactome was strongly biased for proteins annotated to biological pathways typically connected to viral infections like ‘response to virus’ (GO:0009615, 33% of the input genes), ‘immune system process’ (GO:0002376, 49%), ‘localization’ (GO:0051179, 48%), ‘signaling’ (GO:0023052, 73%), or ‘programmed cell death’ (GO:0012501, 40%). It should be noted that this bias may in part be amplified by the preferential selection of baits that were known to influence these pathways. The results of the GO term enrichment analysis are provided in Supplementary Table S3.

**Table 1:** Experimentally identified interactions between ASFV–ASFV and ASFV–host gene products.

Cellular Process	ASFV	Host/ASFV	Molecular Mechanism	Reference
1. ASFV intracellular trafficking				
Cytoplasmic transport	B646L	CD1d	Mediates ASFV entry	[27]
	EP402R	AP1G1	Interacts via the C-terminal domain	[28]
	EP402R	DBNL (SH3P7)	Interacts via the proline repeats with the SH3 domain of DBNL	[29]
	CP204L	VPS39	Interacts with the CHC repeat of VPS39 and blocks its binding to the HOPS complex	[30]
	CP204L	DAB2	-	[31]
	E183L	DYNLL1	Interacts via the SQT motif at the C-terminus	[32]
	E199L	RAB5/7/11	-	[33]
	CP2475L (p34)	RAB5/7/11	-	
	E199L	VAPA/B	-	
	CP2475L (p34)	VAPB	-	
Viral membrane fusion	E199L	LAMP1	-	[34]
	E199L	LAMP2	-	
	E199L	NPC1	Interacts with the C-terminal domain of NPC1	
	E248R	LAMP2	-	
	E248R	NPC1	Interacts with the C-terminal domain of NPC1	
Autophagy	A179L	BECN1	Interacts via BH3 homology domain	[35]
	E199L	PYCR2	Induces autophagy by PYCR2 downregulation	[36]
Others	EP152R	TMEM87A	-	[37]
	MGF360-16R	SDCBP	-	[38]
	MGF360-16R	SERTAD3	-	
	EP152R	SGTA	-	[37]
2. Antiviral immune responses				
cGAS-STING pathway	C129R	2′,3′-cGAMP *	Cleaves 2′,3′-cGAMP	[39]
	EP364R	2′,3′-cGAMP *	Cleaves 2′,3′-cGAMP and inhibits its binding to STING	
	L83L	cGAS/STING	Promotes degradation of cGAS and STING	[40]
	MGF505-3R	cGAS/TBK1/ IRF3	Promotes degradation of TBK1 and inhibits phosphorylation of IRF3 and TBK1	[41]
	MGF505-7R	STING/ULK1	Promotes STING degradation via ULK1-induced autophagy–lysosome pathway	[42]
	D117L	STING	Interacts via N-terminal and middle domain with STING and inhibits its interaction with TBK1 and IKBKE	[43]
	E184L	STING	Inhibits STING oligomerization and STING-TBK1-IRF3 complex formation, leading to retention of IRF3 in the cytoplasm	[44]
	MGF505-11L	STING	Promotes degradation of STING by the lysosomal, ubiquitin-proteasome, and autophagy pathways	[45]
	EP402R	STING	Interacts via the C-terminal domain with the transmembrane domain of STING and prevents STING translocation to Golgi	[46]
	MGF360-11L	TBK1	Interacts via the C-terminal domain and promotes degradation of TBK1	[47]
	MGF505-7R	TBK1	Promotes proteosome-mediated degradation and reduces phosphorylation of TBK1	[48]
	A137R	TBK1	Promotes the autophagy-mediated lysosomal degradation of TBK1, leading to retention of IRF3 in the cytoplasm	[49]
	MGF505-7R	IRF3	Inhibits phosphorylation and blocks nuclear translocation of IRF3	[50]
	E120R	IRF3	Interacts via 72-73aa with IRF3, blocks the interaction of IRF3 with TBK1 and consequently suppresses IRF3 phosphorylation	[51]
	E301R	IRF3	Interacts via the N-terminal domain and blocks nuclear translocation of IRF3	[52]
	M1249L	IRF3	Promotes lysosomal degradation of IRF3	[53]
	MGF360-11L	IRF7	Interacts via the C-terminal domain and promotes degradation of IRF7	[47]
	MGF505-7R	IRF7	Promotes the autophagy- and proteosome-mediated degradation and reduces phosphorylation of IRF7	[48]
	S273R	IKBKE	Mediates IKBKE de-SUMOylation and blocks its interaction with STING	[54]
	I215L	RNF138/ RNF41	Mediates degradation of RNF128 via RNF138 to suppress RNF128-mediated K63-linked polyubiquitination of TBK1	[55]
	MGF360-14L	TRIM21	Promotes degradation of IRF3 via TRIM21-mediated K63-linked ubiquitination	[56]
NF-kB/NFAT pathway	A238L	EP300	Displaces EP300 from the CRE/Ƙ3 complex	[57]
	A238L **	RELA (p65)	Forms A238L-RELA complex and blocks nuclear translocation of RELA	[58,59]
	A238L	PPP3CA	Interacts via the PxIxITxC/S motif and inhibits PPP3CA activity	[60,61]
	D345L	IKKA (CHUK)	Interacts with IKKA and suppresses its kinase activity towards IκBα	[62]
	MGF505-7R	IKKA (CHUK)	-	[50]
	D345L	IKKB (IKBKB)	Interacts with IKKB and suppresses its kinase activity towards IκBα	[62]
	F317L	IKKB (IKBKB)	Reduces phosphorylation of IKKB and suppresses its kinase activity towards IκBα	[63]
	MGF360-12L	KPNA2/3/4	Interrupts the interaction of RELA with importins KPNA2, KPNA3, and KPNA4, leading to retention of RELA in the cytoplasm	[64]
	H240R	NEMO	Interacts with the CC1 domain of NEMO, promotes its degradation, and inhibits NEMO-IKKB binding	[65,66]
	EP402R	CD58	-	[67]
IFNs-activated pathways	EP402R	IFNAR1	Inhibits interaction of IFNAR1 with TYK2	[46]
	EP402R	IFNAR2	Inhibits interaction of IFNAR2 with JAK1	
	MGF505-7R	JAK1	Promotes RNF125-mediated JAK1 degradation	[68]
	MGF505-7R	RNF125	Upregulates RNF125 expression	
	MGF505-7R	JAK2	Inhibits the expression of JAK2 via downregulating the expression of HES5	
	MGF505-7R	HES5	Downregulates HES5 expression	
	MGF505-7R	IRF9	Inhibits IRF9 nuclear translocation, binding to STAT1/2 and ISGF3 trimerization	[69]
	MGF360-9L	STAT2	Degrades STAT2 through the ubiquitin–proteasome pathway	[70]
	MGF360-9L	STAT1	Degrades STAT1 through the apoptotic pathway	
	S273R	DCST1	Mediates K48-linked polyubiquitination at K55 of STAT2, leading to its degradation	[71]
	S273R	STAT2	Enhances interaction between STAT2 and DCST1	
	I215L	STAT2	Induces STAT2 ubiquitination and proteasomal degradation	[72]
	I215L	IRF9	Promotes IRF9 degradation via the autophagy–lysosome pathway	[73]
	EP153R	CSF2RA	Interferes with KAP1-STAT3 binding and promotes the phosphorylation of STAT3 in the nucleus	[74]
NLRP3 inflammasome	H240R	NLRP3	Interacts with NACHT and LRR domains of NLRP3 and inhibits NLRP3 inflammasome assembly	[65,66]
	MGF505-7R	NLRP3	Interacts with NACHT and LRR domains of NLRP3 and inhibits NLRP3 inflammasome assembly	[50]
	L83L	IL1B	Interacts via the FTSE motif at the C-terminus	[75]
Others	I267L	RNF135 (Riplet)	Prevents RNF135 from catalyzing K63-linked polyubiquitination and activation of RIG-I	[76]
	CP204L	OAS1	-	[31]
	CP204L	PARP9	-	
3. Cell death				
Apoptosis	A179L	Bad	-	[77,78]
	A179L	BAK1		
	A179L	BAX	-	
	A179L	BBC3 (PUMA)	-	
	A179L	BIK	-	
	A179L	Bmf	-	
	A179L	Hrk (DP5)	-	
	A179L	P13-tBID	-	
	A179L	P15-tBID	-	
	A179L	Biklk	-	[77]
	A179L	BimEL	-	
	A179L	BimL	-	
	A179L	BimS	-	
	E199L	BCL-XL	Interacts with the BH3 domain of BCL-XL	[79]
	E199L	MCL-1	-	
	E199L	BCL-W	-	
	E199L	BCL-2A1	-	
	A224L	CASP3	Inhibits the proteolytic processing of CASP3	[80]
	EP152R	BAG6	-	[37]
	EP152R	SGTA	-	[37]
Pyroptosis	S273R	GSDMD	Cleaves GSDMD at the site G107-A108	[81]
4. Cellular translation				
	CP204L	HNRNPK	Interacts with KH1 and KH2 domains of HNRNPK	[82]
	D250R	RPL23A	-	[83]
	DP71L	EIF2A, PPP1CA/B/C	Forms a ternary complex DP71L-PPP1C-EIF2A and acts through PPP1C isoforms to dephosphorylate EIF2A	[84]
	E66L	PKR	Interacts via the transmembrane domain with PKR to phosphorylate EIF2A	[85]
	H339R	NACA	-	[86]
	CP204L	RPSA	-	[31]
	CP204L	VBP1	-	
	I215L	CUL4B	-	[87]
	I215L	RPS23	-	
	I215L	EIF4E	-	
5. Virus assembly				
	A151R	E248R	Mediates oxidation of E248R	[88]
	B119L	A151R	-	
	E120R	B646L	Mediates incorporation of E120R into the virus particle	[89]
	EP84R	CP2475L	Interacts with the N-terminal domain of CP2475L and guides the formation of the virus core–shell	[90]
	M1249L	B646L/D117L	Interacts with B646L and D117L and forms the zipper structure, which constructs the capsid framework	[91]
	S273R	CP2475L/CP530R	Cleaves the viral polyproteins CP2475L and CP530R to mature products and intermediate precursors	[92]
6. Other				
	MGF110-7L	PDIA3	-	[93]
	MGF110-7L	PSMA4	-	
	MGF110-7L	TMED4	-	
	MGF360-15R	DDX3	-	[33]
	MGF360-15R	TUBA4A	-	

* 2′3′-Cyclic guanosine monophosphate–adenosine monophosphate (cGAMP). ** 32-kDa post-translationally modified form of A238L. Abbreviations: SH3: SRC homology 3; CHC: clathrin heavy-chain; HOPS: homotypic fusion and vacuole protein sorting; CC: coiled-coil; LRR: leucine-rich-repeat; KH: K homology.

## 3. ASFV Intracellular Trafficking

During the initial stages of ASFV replication, the virus particle attaches to the cell by interacting with a hitherto-unknown surface receptor(s) through its external lipid envelope and is internalized mainly via clathrin- and dynamin-mediated endocytosis [94,95] and macropinocytosis [96]. Previous reports indicated that ASFV virions could effectively infect cells even in the absence of external lipid envelopes [97,98]. In this case, a recent study showed that the virus entry is facilitated by a host protein called CD1d located on the cell surface [27]. Also, the role of CD163 as a likely entry receptor for ASFV has been a topic of several studies. Sánchez-Torres et al. [99] reported that CD163 expression correlated with monocyte-derived cell population susceptibility, and anti-CD163 monoclonal antibodies reduced infection in alveolar macrophages. However, the expression of CD163 in other non-susceptible cell lines did not lead to an increase in susceptibility to ASFV [100], and the CD163 knockout pigs were fully susceptible to ASF [101]. A recent study [102] suggested that SIGLEC1 and CD163 may mediate ASFV entry collaboratively. Specifically, PK15 and 3D4-21 cells showed susceptibility to ASFV infection only when both CD163 and SIGLEC1 were expressed. Further validation is required to confirm the role of SIGLEC1 and CD163 as key receptors involved in ASFV entry.

The transport of internalized particles to perinuclear virus replication factories proceeds along the endolysosomal pathway in a stepwise pH-dependent manner from early endocytic/macropinocytic vesicles to late endocytic compartments. Once the virus particles reach the late endosome, they undergo uncoating, losing their outer envelope and protein capsid. Subsequently, the exposed inner envelope fuses with the late endosomal membrane and the naked cores are released in the cytosol [103,104]. Currently, only a few host protein targets that mediate ASFV trafficking have been identified.

### 3.1. Cytoplasmic Transport

The outer membrane protein EP402R (CD2v) has been found to interact with two adaptor proteins, the clathrin adaptor protein 1 (AP-1) [28] and the actin-binding adaptor protein (DBNL, also called SH3P7) [29], which can modify cellular transport mechanisms and involve Golgi reorganization. Similar to the interaction between Nef and AP-1 in HIV-infected cells, the binding to AP-1 and DBNL may disrupt MHC-I trafficking [105]. Alternatively, these interactions may mediate membrane rearrangement to construct a scaffold for viral replication and morphogenesis, known as a virus factory (VF), since EP402R localizes to the boundaries of the VF. Recently, another ASFV protein, CP204L (p30), was reported to affect the architecture of the VF. Through interaction with VPS39, a component of the homotypic fusion and vacuole protein sorting (HOPS) complex, CP204L mediates the recruitment and clustering of lysosomal membranes at the VF [30]. Meanwhile, the ASFV inner membrane protein E183L (p54) exploits the dynein motor complex by binding with the cytoplasmic dynein’s light chain (DYNLL1), an interaction that is crucial for ASFV transport and localization of viral proteins to the VF [32]. Moreover, both proteins, E199L and CP2475L (p34), interact with several endocytic pathway regulators such as RAB5, RAB7, RAB11, and VAPB, indicating their important function in modulating host intracellular trafficking to promote ASFV entry and replication [33].

In addition to specific PPIs, the formation of VF involves the reorganization of cellular compartments, including mitochondria, ribosomes, and microtubules (reviewed in [106]). Furthermore, ASFV modifies endosomal trafficking and redirects endosomal membranes to the viral replication site [107]. The role of endosomal components in ASFV assembly is unknown, but they may be crucial for virus replication by providing a scaffold and restricting the process to a specific cytoplasmic location.

### 3.2. Viral Membrane Fusion in Endosomes

To date, two ASFV inner membrane proteins, E248R and E199L, have been demonstrated to be crucial for the fusion of viral and cellular membranes and delivery of the genome-containing core into the cytoplasm [103,108]. Recently, Cuesta-Geijo et al. uncovered an interaction between E248R and E199L and specific host proteins, namely, late endosomal cholesterol transporter Niemann Pick C1 (NPC1) and lysosomal membrane proteins (Lamp)-1 and -2 [34]. Notably, certain viruses, including Ebola and Lassa, rely on NPC1 and Lamp proteins for endosomal fusion, and the complete depletion of these host proteins leads to the cessation of viral infection [109,110]. However, in vitro studies suggest that the inhibition of ASFV infection in the absence of NPC1 or Lamp2 is only partial [34], suggesting that the virus might utilize alternative pathways and host proteins to accomplish viral fusion before undergoing lysosomal degradation.

### 3.3. Autophagy

Autophagy is a cellular defense mechanism that can deliver viral components toward lysosomal degradation. Despite its efficacy, viruses have developed strategies to hinder or even exploit autophagy for the benefit of infection.

While it is known that ASFV represses autophagy in infected cells, the precise molecular mechanisms underlying this process are not yet fully elucidated. It has been established that the viral B-cell lymphoma-2 (Bcl-2) homolog, A179L, can suppress autophagosome formation by interacting with Beclin-1 (BECN1) [35]. Notably, an A179L ASFV deletion mutant virus could still repress the formation of autophagosomes, suggesting that A179L-BECN1 interaction contributes to autophagy inhibition, but it is not the sole mechanism utilized by the virus for this purpose [111].

In contrast, ASFV proteins E199L and K205R have been shown to facilitate the induction of cellular autophagy through the interaction with the pyrroline-5-carboxylate reductases (PYCR) or inducing endoplasmic reticulum (ER) stress, respectively [36,112]. Additionally, various ASFV proteins have been found to manipulate autophagy to degrade their cellular binding partners, including TBK1, STING, IRF7, or IRF9. This implies that ASFV evades the innate immune response by co-opting the autophagy pathway.

## 4. Modulation of Innate Immune Signaling Pathways

Interferons (IFNs) are a family of cytokines that can induce an antiviral state in the host by activating the expression of interferon-stimulated genes (ISGs). Based on the similarity of their protein sequences and their receptors, IFNs are divided into three distinct groups, designated as type I (IFN-I), type II (IFN-II), and type III IFNs (IFN-III) [113,114]. Both IFN-I and IFN-II have been demonstrated to inhibit ASFV replication in vitro and in vivo [115,116,117]. Moreover, the attenuated ASFV strains induce higher IFN-I expression in porcine macrophages than virulent strains [118,119]. Together, it suggests that manipulation of IFN-mediated defense contributes to the virulence mechanisms of ASFV.

### 4.1. Evasion of cGAS-STING-Mediated IFN-I Pathway

Many pathogen recognition receptors (PRRs), such as DNA-dependent activator of IFN-regulatory factor (DAI), interferon-gamma inducible protein 16 (IFI16), DDX41, cyclic GMP-AMP synthase (cGAS), and TLR9, have been identified in humans and mice to initiate IFN-I response via sensing DNA. Among those, cGAS emerges as the major cytosolic DNA sensor that triggers IFN-I in response to DNA virus infection [120]. Upon DNA recognition, cGAS produces the second messenger cyclic, GMP-AMP (cGAMP), which binds to and activates STING [121]. Subsequently, STING translocates from ER to Golgi and ER-Golgi intermediate compartment (ERGIC), recruits TBK1, and triggers IRF3 phosphorylation by TBK1, which ultimately activates IFN-I responses [122,123].

It has been well established that ASFV has evolved numerous strategies to impair cGAS-STING-mediated IFN-I responses. In contrast to the virulent Armenia/07, the attenuated NH/P68 strain induces higher activation of STING, TBK1, and IRF3 at the early times of infection, leading to higher expression and production of IFN-β [118]. This is the first evidence demonstrating that the virulent ASFV strain can evade the cGAS-STING pathway. Recently, evidence on the detailed mechanisms by which ASFV manipulates the cGAS-STING pathway has accumulated. By screening the IFN-β promoter activity of 158 ASFV viral proteins, EP364R and C129R were found to inhibit 2′,3′-cGAMP-induced IFN-β activation but not STING expression-activated IFN-β [39]. Importantly, the expression of EP364R or C129R decreases cGAS-STING-mediated production of IFN-I and proinflammatory cytokines and enhances the replication of adenovirus and herpes simplex virus. Mechanistically, EP364R and C129R compete with STING to bind to 2′,3′-cGAMP. Further, EP364R and C129R cleave 2′,3′-cGAMP in vitro and in cells, resulting in the reduction in STING activation and downstream IFN-I signaling [39].

In addition to 2′,3′-cGAMP degradation, ASFV is able to target each signaling component of the cGAS-STING IFN-I pathway via diverse mechanisms, i.e., promoting the degradation or inhibiting their activation. The nonessential protein L83L interacts with cGAS and STING and induces their degradation, which blocks cGAS-STING-mediated IFN-I signaling. Mechanistically, L83L specifically recruits the autophagy receptor TOLLIP and thus triggers the autophagic degradation of STING [40].

Multigene family (MGF)360 and MGF505 genes are critical for the replication of ASFV [124] and the suppression of IFN-I responses in macrophages [125]. MGF505-7R interacts with STING and induces the degradation of STING by autophagy. It also interacts with the serine/threonine protein kinase ULK1, a critical regulator for autophagy initiation, and increases the expression of ULK1. ULK1 knockdown leads to elevated protein levels of STING. Thus, MGF505-7R impairs cGAS-STING signaling by inducing STING degradation, likely via enhanced autophagy mediated by increased ULK1 expression [42]. MGF505-7R also interacts with IRF3 and suppresses its nuclear translocation to block IFN-I production [50]. Additionally, MGF505-7R interacts with IRF7 and TBK1 and subsequently mediates their degradation, thereby inhibiting cGAS-STING-mediated IFN-I signaling [48]. Another member of MGF505, MGF505-11L, specifically interacts with exogenous and endogenous STING and induces the proteasomal and autophagic degradation of STING [45]. The p17 protein encoded by D117L is a major structural protein of the ASFV capsid [126]. As a transmembrane protein, p17 is localized in the ER and Golgi apparatus. It interacts with STING, blocks the recruitment of TBK1 and IKKε, and consequently interferes with the activation of IRF3 and NF-κB during ASFV infection [43]. Likewise, E184L interacts with STING via the N-terminal region (1-20aa) and inhibits the dimerization and oligomerization of STING, thus preventing the expression of IFN-β and IL-1β. Infection of pigs with ASFV ∆E184L induced higher production of IFN-I and IFN-II in vivo, and all pigs survived upon challenge, demonstrating that E184L is a crucial virulence factor [44]. Likely, the product of EP402R, CD2v, also interacts with STING and prevents STING activation by blocking its translocation from ER to Golgi [46].

MGF505-3R also inhibits cGAS-STING-mediated IFN-I signaling. In detail, it interacts with cGAS, TBK1, and IRF3 and inhibits the phosphorylation of TBK1 and IRF3. Additionally, MGF505-3R induces the autophagic degradation of TBK1 [41]. Likewise, both MGF360-11L and MGF505-7R interact with TBK1 and IRF7 and mediate their degradation, leading to impaired IFN-I signaling [47,48]. Protein A137R also interacts with TBK1 and promotes the autophagic degradation of TBK1. Thus, infection with ASFV ∆A137R induces higher IFN-I production in primary porcine alveolar macrophages [49]. As a viral E2 ubiquitin-conjugating enzyme, I215L strongly suppresses IFN-I production mediated by cGAS-STING. Although the interaction between I215L and TBK1 is not detected, I215L significantly inhibits the phosphorylation and RNF128-mediated K63-linked ubiquitination of TBK1, which is required for TBK1 activation [127]. Mechanistically, I215L interacts with the E3 ligase RNF138 and promotes the interaction of RNF138 and RNF128, which in turn leads to the degradation of RNF128 [55]. In addition to TBK1, its homolog IκB kinase (IKK)ε is also required for IRF3 activation during IFN-I induction, although to a lesser extent [128]. ASFV S273R encodes a 31-kDa protein that shows homology with the core domains of SUMO-1-specific proteases and the adenovirus protease. It cleaves the polyproteins pp220 and pp62 to produce mature components of virus particles [129]. It also interacts with IKKε and disrupts the interaction between IKKε and STING via mediating the de-sumoylation of IKKε, finally leading to the impairment of cGAS-STING signaling [54].

E120R, a structural protein of ASFV, interacts with IRF3 via the amino acids at positions 72 and 73 and impairs the phosphorylation of IRF3 due to decreased interaction between IRF3 and TBK1. An ASFV mutant carrying a deletion of these critical two amino acids of E120R fails to interact with IRF3 and induces higher production of IFN-I [51]. As a capsid protein antagonizing IFN-I activation, M1249L also interacts with IRF3 and triggers the degradation of IRF3 via the lysosomal pathway [53]. Intriguingly, ASFV E301R interacts with IRF3 and blocks the translocation of IRF3 to the nucleus [52], and MGF360-14L interacts with the E3 ligase TRIM21 and promotes IRF3 degradation by increasing TRIM21-mediated K63-linked ubiquitination, resulting in diminished IFN-I production [56].

### 4.2. NF-kB/NFAT Pathway

As global transcriptional coactivators, cAMP response element-binding (CREB)-binding protein (CBP) and its homolog p300 interact with NF-κB to drive proinflammatory cytokine expression [130]. ASFV A238L inhibits TNF-α expression by manipulating NF-κB, the nuclear factor of activated T cells (NFAT), and c-Jun transactivation. Its expression in the nucleus displaces the CBP/p300 coactivators from the TNF-α promoter [57]. As an IκB-like protein, A238L interacts with p65 and prevents p65–p50 binding to the promoter [58,59]. Thus, A238L likely directly binds p65 and inhibits NF-κB activation. A238L expression and interaction with calcineurin can also be linked to the NFAT family of transcription factors that activate various immune responses in broad immune cells. Upon different stimuli, intracellular calcium level is increased, and many calmodulin (CaM)-dependent enzymes, including the phosphatase calcineurin, are activated. Calcineurin dephosphorylates NFAT, leading to its nuclear translocation and activation. A238L inhibits the phosphatase activity of calcineurin by interacting with the catalytic subunit [60,61]. The interaction has been mapped to a 14-amino-acid motif (PxIxITxC/S) of A238L [61].

A number of interactions between ASFV proteins and IKK subunits have been reported. D345L, an ASFV-encoded lambda-like exonuclease, interacts with the kinase domain (KD) and helix–loop–helix (HLH) domains of IKKα and the leucine zipper (LZ) domain of IKKβ and subsequently disrupts their kinase activity, thus suppressing cGAS/STING-mediated IFN-β and NF-κB activation. Interestingly, the function of D345L is independent of its exonuclease activity [62]. MGF505-7R also interacts with IKKα, but not with IKKβ, and inhibits NF-κB activation and NF-κB-mediated IL-1β transcription [50]. ASFV F317L interacts with IKKβ and decreases its phosphorylation, subsequently leading to reduced phosphorylation and ubiquitination of IκBα. Consequently, this results in blocked NF-kB activation via enhanced IκBα stabilization. Accordingly, overexpression of F317L suppresses the expression of proinflammatory cytokines and enhances ASFV replication, while knockdown of F317L expression decreases ASFV replication [63]. MGF360-12L interacts with nuclear transport proteins KPNA2, KPNA3, and KPNA4 and interrupts the interaction between p65 and nuclear transport proteins. This leads to the reduced nuclear transport of p50 and p65, and hence NF-κB activation [64]. H240R specifically interacts with a component of the IKK complex, NF-κB essential modulator (NEMO), but not with IKKα or IKKβ. H240R promotes the autophagic degradation of NEMO and suppresses the phosphorylation of p65 and IκBα, resulting in impaired NF-κB activation [65,66]. Furthermore, EP402R present on infected cell membranes or released by ASFV-infected macrophages interacts with CD58, resulting in NF-κB activation and IFN-β induction [67].

### 4.3. IFN-I, IFN-II, and IFN-III Induced Signaling

IFNs induce the expression of various interferon-stimulated genes (ISGs), which activate the antiviral responses of the host [39,39,113,114,131]. IFN-I and IFN-III bind to the type I interferon receptor complex IFNAR1/2 or IL10RB/IFNLR1, respectively, and activate receptor-associated tyrosine kinase 2 (TYK2) and Janus-activated kinase 1 (JAK1), which induce the phosphorylation of signal transducer and activator of transcription 1 (STAT1) and STAT2. Subsequently, this leads to the formation of STAT1-STAT2-IRF9 complexes (also known as ISGF3). The complexes further translocate to the nucleus and bind IFN-stimulated response elements (ISREs) in the promotor region to initiate the transcription of ISGs. IFN-II binds to its receptor complex IFNGR1/2 and activates the associated JAK1 and JAK2, resulting in the phosphorylation and homodimerization of STAT1 and the translocation of the homodimer into the nucleus. STAT1 homodimers bind to IFN-γ-activated sequence (GAS) elements in the promoters to trigger the transcription. These signaling pathways are essential for IFNs-mediated antiviral responses; hence, many viruses develop diverse strategies to manipulate IFNs-activated signaling pathways. ASFV EP402R interacts with the IFN-I receptors IFNAR1 and IFNAR2 and inhibits IFN-I mediated signaling by impairing the formation of the IFNAR1-TYK2 and IF-NAR2-JAK1 complexes, respectively. Consistently, pigs infected with ASFV ∆EP402R produce higher levels of IFN-β and show higher survival rates [46]. MGF505-7R impacts IFN-induced signaling pathways by establishing a number of PPI with constituents involved in these pathways. It interacts with JAK1 and JAK2 and promotes their proteasomal degradation, leading to impaired IFN-γ signaling. Mechanistically, MGF505-7R interacts with the E3 ligase RNF125 and increases its expression, resulting in RNF125-mediated degradation of JAK1. MGF505-7R also interacts with HES5 and decreases its expression, in turn leading to JAK2 degradation [68]. MGF505-7R also interacts with IRF9, blocking the formation of the ISGF3 complex and its nuclear translocation. ASFV-∆MGF505-7R partially restores the nuclear translocation of ISGF3 and shows increased susceptibility to IFN-β [69]. Another ASFV protein, MGF360-9L, interacts with STAT1 and STAT2, promoting their degradation via apoptosis and proteasome pathways, respectively [70]. ASFV S273R interacts with STAT2 and induces its proteasomal degradation. In contrast to its effect on IKKε [54], S273R-stimulated STAT2 degradation is independent of its protease activity. In detail, S273R recruits the E3 ubiquitin ligase DCST1, consequently leading to K48-linked polyubiquitination of STAT2 and subsequent proteasomal degradation [71]. Similarly, I215L, an E2 ubiquitin conjugation enzyme of ASFV, interacts with STAT2 and promotes its ubiquitination and degradation. The ubiquitination and degradation of STAT2 interaction depend on the catalytic activity of I215L, yet the E3 ligase involved in I215L-mediated STAT2 degradation has not been identified [72].

### 4.4. NLRP3 Inflammasome Activation

Inflammasomes are multiprotein oligomers that are initiated upon sensing danger-associated molecular patterns (DAMPs) or pathogen-associated molecular patterns (PAMPs) by cytosolic pattern recognition receptors (PRRs). The PRRs involved in inflammasome activation include NLRs, i.e., NLRP3 and AIM2. Upon activation, PRRs assemble with the adaptor protein ASC and caspase-1 to promote the activation of caspase-1. Consequently, active caspase-1 triggers the maturation and secretion of proinflammatory cytokines IL-1β and IL-18. Additionally, upon cleavage by active caspase-1, N-terminal fragments of GSDMD rapidly associate with the plasma membrane and form pores, leading to lytic cell death—pyroptosis. The cellular DAMPs such as ATP, mitochondrial DNA, and HMGB1 released by the pyroptotic cells trigger further immune activation and amplify the inflammation [132,133]. It is evident that inflammasome activation can suppress the replication of various viruses during infection, and viruses have evolved evasion mechanisms to overcome inflammasome-mediated immune responses [134]. As a structural protein in the capsid, H240R interacts with NLRP3 and blocks NLRP3 inflammasome activation, as evidenced by the decreased formation of ASC specks and NLRP3 oligomers. Infection with ASFV ∆H240R in porcine alveolar macrophages (PAMs) produces higher IL-1β. NLRP3 knockdown in porcine alveolar macrophages (PAMs) promotes the replication of ASFV ∆H240R but not ASFV WT, demonstrating the antiviral effect of the NLRP3 inflammasome. In vivo challenge with ASFV ∆H240R results in lower pathology and virus replication and higher production of inflammatory cytokines, confirming H240R as a critical virulence factor by inhibiting the NLRP3 inflammasome [65,66]. MGF505-7R also interacts with NLRP3 via its NACHT and LRR domains, and MGF505-7R expression impairs ASC speck formation induced by the NLRP3 inflammasome. Accordingly, in vivo infection with ASFV ∆MGF505-7R leads to elevated IL-1β production and compromised pathology and virus replication [50]. ASFV L83L interacts with IL-1β; however, ASFV ΔL83L is as virulent as the parental virus. Thus, the physiological relevance of the interaction between L83L and IL-1β needs to be further elucidated [75].

### 4.5. Miscellaneous Immune Pathways

ASFV induces RIG-I-mediated IFN-I immune responses. The ASFV genome contains numerous AT-rich regions which activate the RNA Pol-III-RIG-I axis [76]. Intriguingly, I267L directly binds to the E3 ligase Riplet. Riplet catalyzes K63-linked polyubiquitination of RIG-I, which is required for RIG-I activation. Overexpression of I267L compromises the interaction between RIG-I and Riplet, subsequently impairing Riplet-catalyzed K63-linked polyubiquitination and RIG-I-mediated IFN-I signaling. Consistently, ASFV ∆I267L induces higher levels of IFN-β and displays impaired replication both in primary macrophages and pigs [76].

## 5. Cell Death

Viral infections can elicit diverse forms of programmed cell death in the host. Premature cell death precludes the establishment of persistent infection and reduces virus progeny production. However, cell death induction at late infection stages can enhance viral dissemination. Therefore, viruses have developed intricate mechanisms to either block or promote the host cell death machinery at different stages of the infection.

### 5.1. Apoptosis

ASFV triggers apoptosis during the early stages of infection, but significant apoptosis within infected cells occurs primarily at later stages [135,136,137]. So far, only a few ASFV antiapoptotic proteins have been identified, and these are A179L, A224L, EP153R, and DP71L. Previous studies have demonstrated that the early expressed ASFV protein A179L has a broad affinity for the major death-inducing Bcl-2 proteins found in mammals, such as BAX and BAK, Bim, BID, Bad, BIK, Bmf, Hrk, and PUMA, except for NOXA [77,78]. Interestingly, this promiscuity in ligand binding is speculated to be an ASFV adaptation to subvert intrinsic apoptosis machinery in mammals and arthropods. The precise mechanism by which A179L inhibits apoptosis is not yet fully understood. Nonetheless, it is possible that A179L, found in both the mitochondria and ER, hinders the activation and/or oligomerization of proapoptotic proteins Bax and Bak, which are responsible for forming pores in the membranes. This prevents the release of apoptogenic factors from the ER and mitochondrial lumen into the cytosol, the subsequent activation of caspase-9 and caspase-12, and ultimately cell death [138,139].

Downstream of caspase-9, the binding of the A224L protein to the proteolytic fragment of caspase-3 blocks the activation of several proapoptotic proteins, thereby inhibiting the execution of apoptosis [80]. Additionally, A224L induces NF-κB signaling, promoting the expression of antiapoptotic genes [140]. Another viral protein, DP71L, has been observed to regulate global protein synthesis and inhibit transcriptional activation of the proapoptotic CCAAT/enhancer-binding protein homologous protein (CHOP) [84]. This is achieved by targeting protein phosphatase 1 (PP1) to dephosphorylate the translation initiation factor 2α (eIF2α). The ASFV protein EP153R also limits apoptosis by inhibiting the activation of the p53 protein; however, the exact mechanism of activation remains elusive [141,142].

A cytopathic effect can be observed in cultured cells approximately 12 h after infection [143]. The ASFV late protein E199L was reported to interact with several antiapoptotic Bcl-2 family members, such as BCL-XL, BCL-W, BCL-2A1, and MCL-1, leading to activation of the mitochondrial apoptotic pathway [79]. E199L inhibits BAK binding toBCL-XL, thus promoting BAK homooligomerization and subsequently leading to mitochondrial outer membrane permeabilization (MOMP). Furthermore, the structural protein E183L (p54) may induce apoptosis in the later stage of virus infection by binding with DYNLL1 to facilitate BIM translocation to mitochondria [32,144].

Notably, the protein E152R can interact with BAG6 and its co-chaperone SGTA. BAG6 helps maintain cellular homeostasis by monitoring and degrading misfolded or unfolded proteins in the ER. Studies showed that under conditions of ER stress, BAG6 is converted to an autophagy modulator and apoptosis trigger [145]. Given that maintaining a balance between cell death and survival is crucial in preventing diseases, further investigation into the role of BAG6 in the pathogenesis of ASF could help identify potential therapeutic strategies.

### 5.2. Necroptosis and Pyroptosis

Until recently, apoptosis was considered the only molecularly regulated mechanism of cell death with a critical role in responding to viral infections. However, it has now been recognized that necroptosis and pyroptosis are also associated with viral infections and can be activated by pathogens and host molecules. Unlike apoptosis, programmed necrotic cell death is characterized by the rapid loss of plasma membrane integrity, which drives inflammation and immune responses. Necroptosis and pyroptosis are executed by the mixed-lineage kinase domain-like (MLKL) proteins and gasdermin D (GSDMD), respectively. It is important to note that apoptosis, necroptosis, and pyroptosis are interconnected and capable of cross-regulating one another (reviewed in [146]).

In a recent study, Shu et al. demonstrated that ASFV can induce necroptosis [147]. Interestingly, the viral protein A179L was observed not only to inhibit apoptosis but also to promote necroptosis. The A179L protein, expressed at various stages of ASFV infection, appears to have a dual function: it inhibits apoptosis to support viral replication and enhances necroptosis, potentially aiding virus release and spread later in the infection cycle.

The ASFV protease S273R was found to interact with GSDMD, an executor of pyroptosis. During pyroptosis initiation, caspase-1 cleaves GSDMD at the G279 site to yield a cytotoxic pore-forming protein. ASFV S273R cleaves GSDMD at a different site, G107, generating a nonfunctional product [81]. This interaction can potentially hinder the progression of pyroptosis in the infected cells.

Further research is needed to fully comprehend how ASFV controls cell death pathways throughout infection, highlighting the importance of investigating the spatiotemporal regulation of cell death events during viral infection.

## 6. Host Translational Machinery Control of ASFV

The synthesis of viral proteins necessary for replication is fully dependent on the translation machinery of the host cell. Consequently, many virus-encoded proteins are dedicated to hijacking the cellular translation apparatus and manipulating host signaling pathways for optimizing viral gene expression. Recently, the ASFV-induced host cell shutoff (vhs) has been characterized in a proteomic study indicating that the impact of the ASFV infection on the expression of the over 2000 proteins for which the synthesis rates were measured varies over a very broad range [148]. Although our understanding of the mechanisms employed by ASFV to suppress or induce host protein synthesis in infected cells remains limited, a few strategies have been elucidated.

Firstly, ASFV infection causes the relocation and concentration of the host’s translation machinery in viral replication compartments, resulting in the preferential translation of viral mRNA accumulated at the viral replication foci over host mRNA diffused in the cytoplasm [149]. The mechanism by which protein synthesis resources are relocated to the viral factories remains unclear.

Secondly, during ASFV infection, the eukaryotic initiation factor 4F (eIF4F) complex accumulates within viral factories and is activated to enhance the initiation of viral mRNA translation. It has been suggested that the assembly of the eIF4F complex during ASFV infection is regulated by the activation of several factors, including the kinase mTOR complex 1 (mTORC1), Myc proto-oncogene protein (c-Myc) [150], and mitogen-activated kinase 1 (Mnk-1) phosphorylation [149]. ASFV controls the expression of eIF4F complex components during both the early and late stages of infection. Early in infection, an ASFV ubiquitin-conjugating enzyme (I215L) binds to eIF4E, inducing its overexpression, and to cullin 4B (CUL4B), which regulates mTOR activity [87]. At late times of infection, the IAP homolog ASFV protein A224L induces the expression of eIF4G1 and eIF4E subunits of the eIF4F complex [87,150].

A third mechanism of ASFV translational control involves modulation of the translation initiation factor 2α (eIF2α) activity. It has been shown that the viral protein DP71L recruits the cellular phosphatase PP1 to dephosphorylate eIF2α and prevents the inhibition of global protein synthesis [84]. On the other hand, the transmembrane domain of the E66L protein can interact with protein kinase R (PKR), leading to the activation of eIF2α phosphorylation and induction of translational arrest [85]. Similarly, MGF110-7L enhances eIF2α phosphorylation, resulting in host translation suppression. Supposedly, by inducing ER stress, MGF110-7L activates the kinases PERK and PKR, which contribute to the phosphorylation of eIF2α. Cellular proteins PDIA3, PSMA4, and TMED4 were identified as MGF110-7L protein interactors; however, their roles in MGF110-7L-induced stress and eIF2α phosphorylation remain unclear [93].

Finally, ASFV infection leads to the degradation of host cellular mRNAs, which may occur through the action of a viral mRNA decapping enzyme known as D250R (g5R) [83]. In addition, the retention of cellular mRNAs in the nucleus is facilitated by a small RNA-binding protein, I73R [151]. Other PPIs between ASFV proteins and host translation factors have been documented, including those involving ribosomal proteins (RPL23A [83], RPS23 [87], RPSA [31]), ribosome-associated proteins (NACA [86], VBP1 [31]), and mRNA-binding protein (HNRPK [82]). Further understanding of the functional roles of these interactions could offer valuable insights into the complex mechanisms involved in inhibiting host protein synthesis and enhancing viral gene translation.

## 7. Virus Morphogenesis

Advanced cryo-electron microscopy techniques have recently been utilized to examine the intricate and multilayered structure of ASFV, uncovering the composition of five concentric layers: an outer lipid membrane, an icosahedral protein capsid, an inner lipid membrane, a thick protein core–shell, and a nucleoid containing the viral DNA [91,91,152,153]. The latest insights into the capsid structure of ASFV indicate that the previously uncharacterized protein M1249L interacts with both minor and major capsid proteins, D117L (p17) and B646L (p72), respectively. These interactions play a crucial role in forming the capsid framework and determining the capsid’s overall size [91].

ASFV intracellular particles are assembled inside perinuclear viral factories, utilizing viral DNA, proteins, and precursor membranes derived from the ER as fundamental building blocks. The correct assembly was shown to depend on the expression of the viral inner envelope proteins D117L (p17) [126] and E183L (p54) [154], the major capsid protein B646L (p72) and its chaperone B602L [155], the minor capsid protein B438L (p49) [156], and polyproteins CP2475L (pp220) [157] and CP530R (pp62) [158], whose proteolytic products are components of the core–shell.

Some direct interactions between viral proteins have been identified as critical for the formation of ASFV particles. An interaction between E120R and B646L (p72) mediates ASFV capsid assembly. While E120R is not essential for forming infectious intracellular virions, its inhibition drastically reduces the number of extracellular ASFV by impairing the transport of viral particles from the assembly sites to the plasma membrane [89]. The recently characterized inner envelope protein, EP84R, guides the formation of the core–shell by interacting with the polyprotein precursor CP2475L (pp220) [90]. Concomitantly, protein precursors CP2475L (pp220) and CP530R (pp62) are processed by the ASFV protease S273R, and their proteolytic products build the viral core–shell. This process is crucial for the generation of infectious particles [92].

Furthermore, ASFV encodes components of a redox pathway that enables the formation of disulfide bonds in viral proteins within the reducing environment of the cytosol. E248R, an inner envelope protein containing intramolecular disulfide bonds, has been identified as a substrate for this viral redox pathway [88]. The oxidation of E248R is catalyzed by A151R and initiated by the viral FAD-linked sulfhydryl oxidase, B119L, which is the first component of this pathway. As the deficiency of B119L, but not E248R, has a strong impact on the morphology of ASFV, it is possible that the virus’s sulfhydryl oxidases target other structural proteins before they are incorporated into virions [103,159].

## 8. Conclusions and Perspectives

The exploration of ASFV–host protein interactions has come a long way since the first paper in 1998 that reported an experimentally defined ASFV–host protein–protein interaction. In this work, we summarize current knowledge of protein interactions involving ASFV and its host, as well as the interactions among viral proteins themselves. We retrieved 118 protein–protein interactions from the literature and discussed their molecular mechanisms and importance in establishing viral infection and evading host immune responses. To our knowledge, this is the first such comprehensive evaluation of ASFV–host protein–protein interactions, from which several important conclusions can be drawn.

**Figure 2:**
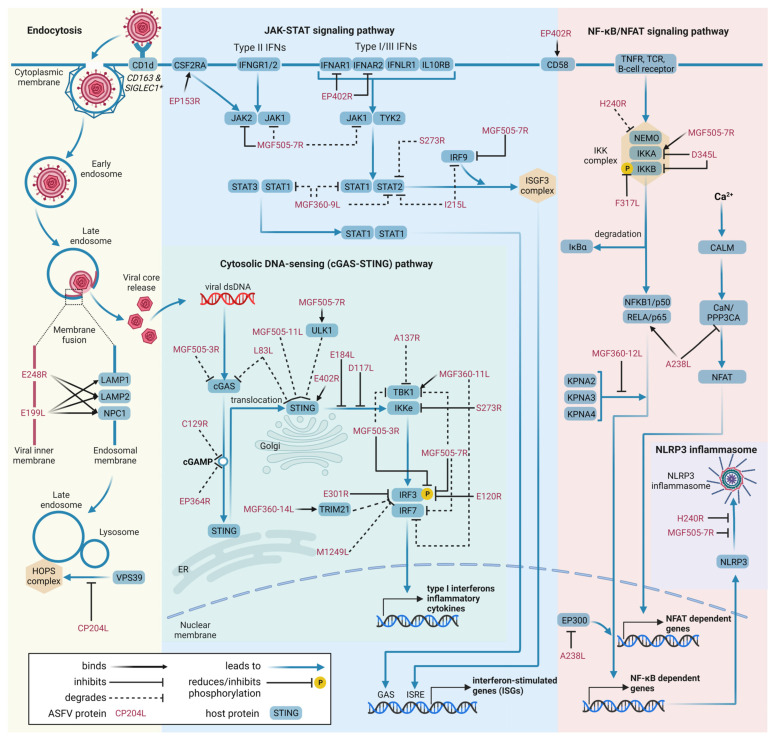
Schematic overview of cellular pathways modulated by ASFV. The pathways have been reproduced and simplified from KEGG pathway maps [160] for endocytosis (hsa04144), cytosolic DNA-sensing (cGAS-STING) pathway (hsa04623), JAK-STAT signaling pathway (hsa04630), and NF-ĸB (hsa04064) and NFAT (hsa04660) signaling pathways. Viral proteins are marked in red font, and host proteins are in blue boxes. The effects caused by the interactions between proteins are represented by the edges and explained in the legend. Created with BioRender.com. * CD163 and SIGLEC1 are considered to act together as potential receptors for ASFV entry.

Firstly, of the more than 150 ORFs encoded by ASFV, only 49 viral proteins have been reported to interact with one or more proteins. Thus, it is necessary to expand the search for interacting partners for the remaining 100 ASFV proteins, particularly those that remain uncharacterized. Another important aspect emphasized by this review is the overlapping functions among viral proteins. Multiple proteins encoded by ASFV exhibit binding to common cellular targets, with a significant proportion of these targets belonging to the cGAS-STING, JAK-STAT, or NF-κB signaling pathways (Figure 2). At the same time, viral proteins display multifunctionality by engaging with diverse host factors. Given the intricate replication cycle of ASFV, which includes the temporal regulation of gene expression and transcription, the viral proteins may display stage-specific activity and acquire novel functions to facilitate certain steps throughout the replication process. Additionally, exploring the interactions between ASFV proteins and various suid and arthropod hosts can enhance our understanding of viral evolution, replication, and long-term persistence.

Future studies should address these challenges by analyzing ASFV–host interactions at different stages of infection and in different host cells, including hosts representing the arthropod vectors as well as representatives of the *suidae*, which show different susceptibility to ASFV infection. Moreover, global and unbiased analysis of virus–host protein interactions based on high-throughput techniques such as affinity purification mass spectrometry is needed for the identification of novel host factors and pathways involved in ASFV infection and pathogenesis. Here, the systematic comparison of the interactomes of ASFV proteins from strains with different virulence could be helpful to identify PPIs that potentially are relevant virulence determinants. Finally, establishing a systematic and easily accessible repository of knowledge on ASFV–host PPIs would offer a comprehensive platform to build upon, ultimately accelerating the development of antiviral drugs in the field.

## Supplementary Materials

The following supporting information can be downloaded at: https://www.mdpi.com/article/10.3390/v15081634/s1, Figure S1: Network based exclusively on published ASFV–protein interactions; Table S1: List of published ASFV–host PPIs including detailed information about the bait, prey, and experimental method used in the study; Table S2: Results of the network analysis performed with the cytoHubba plugin for Cytoscape; Table S3: Results of the GO term enrichment analysis performed with gProfiler; Supplementary Methods File S1: Description of statistical methods [161].
