# Supplementary material for: Functional Landscape of African Swine Fever Virus–Host and Virus–Virus Protein Interactions
Source: Viruses. 2023 Jul 27;15(8):1634. doi: 10.3390/v15081634 (PMC10459248; doi:10.3390/v15081634)
Supplement: Supplementary file 1 [file viruses-15-01634-s001.zip › Supplemetary Methods File S1_revised.pdf]

Supplementary Methods File S1 (Statistical methods)

Functional Landscape of African Swine Fever Virus-Host and Virus-Virus Protein Interactions

Katarzyna Magdalena Dolata [1,*], Gang Pei [2], Christopher L. Netherton[3], Axel Karger [1,*]

Statistical Methods

Tables with the interaction data from the literature from Supplementary Table S1 and with the metadata (ASFV protein features) were processed with R-scripts [1] for the following GO-term enrichment analysis with the R package gProfiler2 [2] and the automated construction of the networks with Cytoscape software [3] using the R package RCy3 [4]. Cytoscape StringApp [5] was used for the addition of physical protein-protein interaction from the String database [6].

The main steps of the script were (1) reformatting of the tables to meet the requirements of the RCy3 package for automated network construction with Cytoscape, (2) definition of Cytoscape styles and layouts, (3) triggering the addition of physical PPI from the String database using the StringApp with a minimum confidence score cutoff ≥ 0.5, and without addition of secondary interactions, and (4) mapping of the gene identifiers for term-enrichment analysis with gProfiler2. Analysis of the network was carried out with the CytoHubba [7] plugin for Cytoscape.

The gProfiler query (GO database) was carried out on basis of the HGNC symbols and the species was set to 'Human' as the Gene Ontology data for swine is incomplete. Supplementary Table S3 contains the standard output of gProfiler which displays the adjusted *p*-values. A maximum *p*-value of 0.05 was chosen together with false discovery rate (FDR) computation as the 'correction_method' parameter. The standard set of available databases.

The PPI-enrichment *p*-value (line 83 of the revised manuscript) is an output of the StringApp. The *p*-value refers to testing the number of edges in the network against the number expected for a network with the same number of nodes which were randomly drawn from the genome.

References

1. R Core Team. R: A language and environment for statistical computing, 2020. Available online: https://www.r-project.org.
2. Kolberg, L.; Raudvere, U.; Kuzmin, I.; Vilo, J.; Peterson, H. gprofiler2 -- an R package for gene list functional enrichment analysis and namespace conversion toolset g:Profiler. *F1000Res.* **2020**, *9*, doi:10.12688/f1000research.24956.2.
3. Shannon, P.; Markiel, A.; Ozier, O.; Baliga, N.S.; Wang, J.T.; Ramage, D.; Amin, N.; Schwikowski, B.; Ideker, T. Cytoscape: a software environment for integrated models of biomolecular interaction networks. Genome Res. 2003, 13, 2498–2504, doi:10.1101/gr.1239303.
4. Gustavsen, J.A.; Pai, S.; Isserlin, R.; Demchak, B.; Pico, A.R. RCy3: Network biology using Cytoscape from within R. F1000Res. 2019, 8, 1774, doi:10.12688/f1000research.20887.3.

5. Doncheva, N.T.; Morris, J.H.; Holze, H.; Kirsch, R.; Nastou, K.C.; Cuesta-Astroz, Y.; Rattei, T.; Szklarczyk, D.; Mering, C. von; Jensen, L.J. Cytoscape stringApp 2.0: Analysis and Visualization of Heterogeneous Biological Networks. J. Proteome Res. 2023, 22, 637–646, doi:10.1016/j.actatropica.2016.03.031.
6. Szklarczyk, D.; Kirsch, R.; Koutrouli, M.; Nastou, K.; Mehryary, F.; Hachilif, R.; Gable, A.L.; Fang, T.; Doncheva, N.T.; Pyysalo, S.; et al. The STRING database in 2023: protein-protein association networks and functional enrichment analyses for any sequenced genome of interest. Nucleic Acids Res. 2023, 51, D638-D646, doi:10.1093/nar/gkac1000.
7. Chin, C.-H.; Chen, S.-H.; Wu, H.-H.; Ho, C.-W.; Ko, M.-T.; Lin, C.-Y. cytoHubba: identifying hub objects and sub-networks from complex interactome. BMC Syst. Biol. 2014, 8 Suppl 4, S11, doi:10.1186/1752-0509-8-S4-S11.
