# Supplementary figures and images for: Functional Landscape of African Swine Fever Virus–Host and Virus–Virus Protein Interactions
Source: Viruses. 2023 Jul 27;15(8):1634. doi: 10.3390/v15081634 (PMC10459248; doi:10.3390/v15081634)

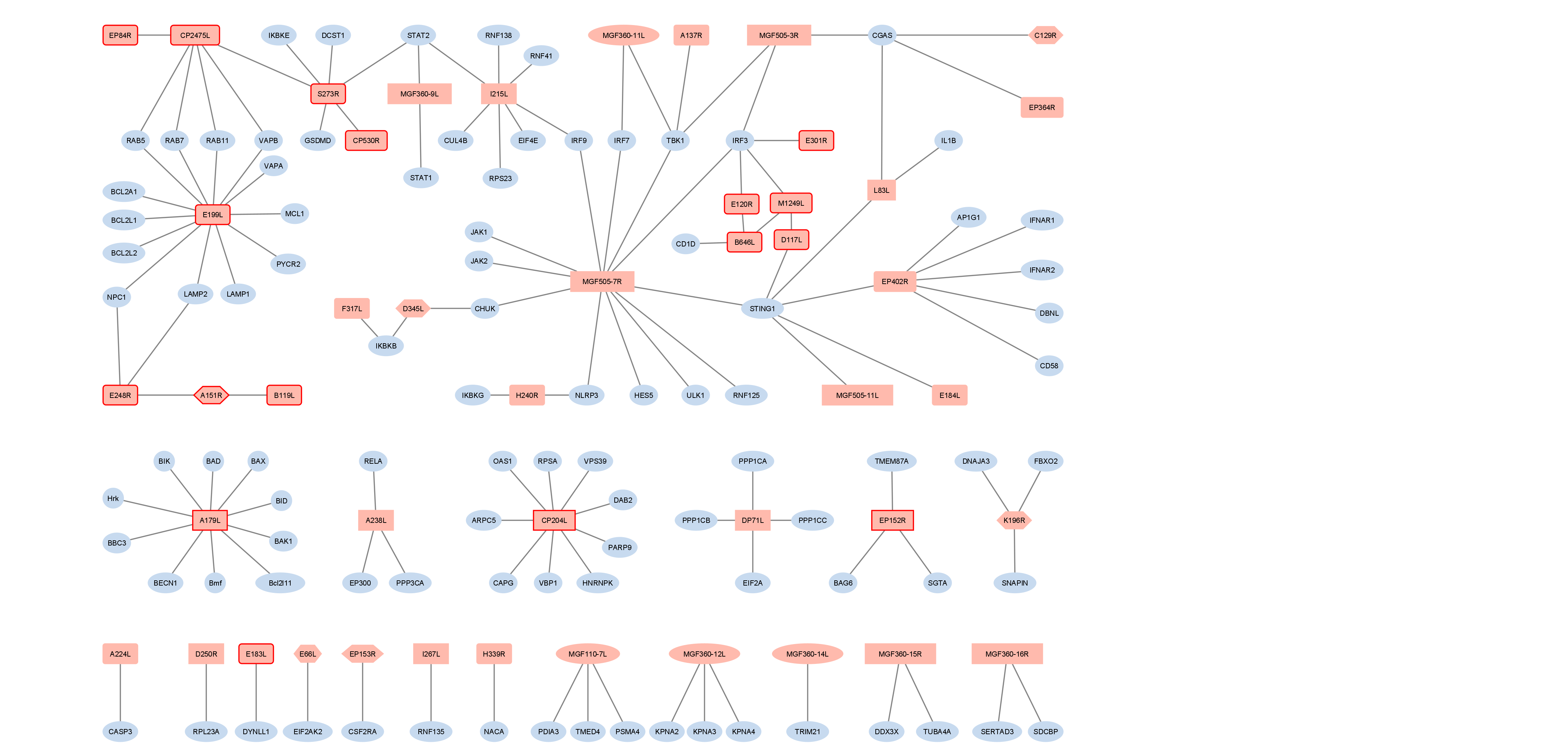

Supplement: Supplementary file 1 [file viruses-15-01634-s001.zip › Supplementary_Figure_S1_revised.png]
